# A New Activity Assay Method for Diamine Oxidase Based on Matrix-Assisted Laser Desorption/Ionization Time-of-Flight Mass Spectrometry

**DOI:** 10.3390/molecules29204878

**Published:** 2024-10-14

**Authors:** Jan Strnad, Miroslav Soural, Marek Šebela

**Affiliations:** 1 Department of Biochemistry, Faculty of Science, Palacký University, Šlechtitelů 27, CZ-779 00 Olomouc, Czech Republic; jan.strnad@upol.cz; 2 Department of Organic Chemistry, Faculty of Science, Palacký University, 17. listopadu 12, CZ-771 46 Olomouc, Czech Republic; miroslav.soural@upol.cz

**Keywords:** activity assay, amine oxidase, enzyme kinetics, MALDI, polyamine, reaction rate

## Abstract

Copper-containing diamine oxidases are ubiquitous enzymes that participate in many important biological processes. These processes include the regulation of cell growth and division, programmed cell death, and responses to environmental stressors. Natural substrates include, for example, putrescine, spermidine, and histamine. Enzymatic activity is typically assayed using spectrophotometric, electrochemical, or fluorometric methods. The aim of this study was to develop a method for measuring activity using matrix-assisted laser desorption/ionization time-of-flight (MALDI-TOF) mass spectrometry based on the intensity ratio of product to product-plus-substrate signals in the reaction mixtures. For this purpose, an enzyme purified to homogeneity from pea (*Pisum sativum*) seedlings was used. The method employed α-cyano-4-hydroxycinnamic acid as a matrix with the addition of cetrimonium bromide. Product signal intensities with pure compounds were evaluated in the presence of equal substrate amounts to determine intensity correction factors for data processing calculations. The kinetic parameters *k*_cat_ and *K*_m_ for the oxidative deamination of selected substrates were determined. These results were compared to parallel measurements using an established spectrophotometric method, which involved a coupled reaction of horseradish peroxidase and guaiacol, and were discussed in the context of data from the literature and the BRENDA database. It was found that the method provides accurate results that are well comparable with parallel spectrophotometry. This method offers advantages such as low sample consumption, rapid serial measurements, and potential applicability in assays where colored substances interfere with spectrophotometry.

## 1. Introduction

Copper amine oxidases (CAOs; EC 1.4.3.21 and 1.4.3.22) are enzymes that catalyze the oxidative deamination of primary amines to the corresponding aldehyde, ammonia, and hydrogen peroxide. As a specific group, diamine oxidases (DAOs; EC 1.4.3.22) oxidize, for example, the diamines putrescine and cadaverine, the polyamines spermidine and spermine, and histamine [1]. CAOs are found in various living organisms, including bacteria, fungi, plants, and animals [2]. They play important roles in the catabolism of biogenic amines, regulation of polyamine levels, and compartment-specific production of hydrogen peroxide. They are implicated in growth, developmental, and defense processes [2,3]. CAOs typically contain a mononuclear type-2 (“non-blue”) copper (II) complex, coordinated by three conserved histidine residues and one/two water molecules [4]. Additionally, the active site houses a unique cofactor 2,4,5-trihydroxyphenylalanine quinone, i.e., topaquinone (TPQ). Both these active-site components are essential for catalysis [5,6], which also involves the proton-abstracting catalytic base, a conserved aspartate residue [7]. The cofactor is derived from an oxygen-dependent autocatalytic post-translational modification of a specific tyrosine residue within the consensus sequence Thr-X-X-Asn-Tyr-Asp/Glu [4,8].

Catalysis in CAOs follows a ping-pong mechanism, consisting of the following two half-reactions: reductive and oxidative (Equations (1) and (2), respectively) [9,10]. First, a primary amine substrate is oxidized, yielding the product aldehyde by hydrolysis. The C5 carbonyl group of TPQ is the site of nucleophilic attack by the amine, resulting in the reduction of the cofactor to its aminoquinol form. In the oxidative half-reaction, the aminoquinol is reoxidized, which is accompanied by the reduction of O_2_ to H_2_O_2_ and the release of NH_4_^+^ [4]. The reduced TPQ exists in equilibrium between the aminoquinol/Cu(II) form and the semiquinone radical/Cu(I) species [11]. This equilibrium is different for CAOs of a particular origin (plant vs. non-plant enzymes). Finally, electrons are transferred to oxygen, forming hydrogen peroxide, while the iminoquinone cofactor releases the ammonium product. This step may occur in two modes of the first electron transfer, either involving the copper (I) ion or not, as proposed and demonstrated in different CAOs [4]. Several catalytic intermediates have been characterized by X-ray crystallography [12,13,14,15].

E + RCH_2_NH_3_^+^ → E•RCH_2_NH_3_^+^ → E_red_ + RCHO (1)

E_red_ + O_2_ → E + H_2_O_2_ + NH_4_^+^ (2)

Pea (*Pisum sativum*) seedling amine oxidase (PSAO) is one of the best-characterized examples of plant DAOs [10,16,17]. It is a homodimer with a subunit molecular mass of 72 kDa [17]. PSAO activity levels are particularly prominent during the early stages of seedling growth and development [18]. This suggests that PSAO plays a role in processes related to germination and early plant growth. In plants, DAO has been implicated in cell wall maturation. It plays a role in the cross-linking of cell wall components, contributing to the strengthening and rigidity of plant cell walls. Cellular levels of DAO can be influenced by various environmental stresses, including pathogen attack, wound healing, and salinity [19]. The substrate specificity of CAOs is determined by the dimensions and chemical composition of the substrate/product channel, as well as the conformation of the substrate–Schiff base complex [4]. PSAO exhibits broad specificity, oxidizing C_4_–C_6_ diamines, spermidine, spermine, agmatine, histamine, tyramine, phenylethylamine, and many other substrates [16,20].

CAOs have potential applications in biotechnology, particularly in the development of biosensors and in the synthesis of specific aldehydes through the selective oxidation of amines [21,22]. Various analytical methods can be used to assess DAO activity, with spectrophotometric assays being the most common. These assays rely on the colorimetric detection of the reaction product (typically an aldehyde or H_2_O_2_) using a specific reagent and monitoring the increase in absorbance over time. The product of putrescine oxidation, 4-aminobutanal, spontaneously cyclizes to 1-pyrroline, which then reacts with 2-aminobenzaldehyde to form a yellow quinazolinium absorbing at 430 nm [23]. In continuous measurements, hydrogen peroxide is detected using a coupled peroxidase reaction with reagents such as guaiacol [24], 2,2′-azino-di-(3-ethylbenzothiazoline-6-sulphonic acid) [25], or 4-aminoantipyrine and 3,5-dichloro-2-hydroxybenzenesulfonic acid [25,26]. Colored products are formed (λ_max_ in the range of 400–600 nm). These methods cannot be used in the presence of catalase, as it decomposes the generated H_2_O_2_, thus affecting the measurement [27]. An alternative amperometric method involves measuring the rate of oxygen consumption in the reaction using an oxygen electrode [28]. A highly sensitive luminometric assay is based on the horseradish peroxidase-catalyzed oxidation of luminol by H_2_O_2_ produced in the DAO reaction [29]. High-performance liquid chromatography can also be used to separate and quantify products resulting from the oxidation of fluorogenic and chromogenic reagents, offering high sensitivity and accuracy [30].

The choice of an assay method depends on the required sensitivity, specificity, ease of use, and equipment availability. Matrix-assisted laser desorption/ionization time-of-flight mass spectrometry (MALDI-TOF MS) has consistently proven to be a powerful approach for conducting enzyme activity assays [31]. This analysis is based on detecting signals from both the substrate and product in the reaction mixture. The substrate conversion rate is determined by calculating the product-to-substrate peak intensity or area ratio or by using a suitable internal standard. MALDI-TOF MS has been successfully used in activity assays of enzymes from different classes, including, among others, hydrolases [32,33] or oxidoreductases such as glucose oxidase [34] or isocitrate dehydrogenase [35]. In principle, it is applicable to the analysis of substrate conversions accompanied by a mass change. The necessary prerequisites for MALDI-based assays are the presence of detectable and distinguishable substrate and product signals, allowing for the measurement of their intensity ratios [32], or capture of the reaction product, e.g., using functionalized self-assembled monolayers [35]. Product quantification is made accurate by utilizing an appropriate internal standard, with isotopically labeled compounds commonly employed for this purpose [34,35]. In this study, MALDI measurements were used to analyze the oxidative deamination of natural and synthetic substrates by PSAO. Alpha-cyano-4-hydroxycinnamic acid (CHCA) served as the matrix compound, with cetrimonium bromide (CTAB) as an additive [36]. The obtained MS results were compared with those from an established spectrophotometry involving horseradish peroxidase and guaiacol.

## 2. Results

**Figure 1:**
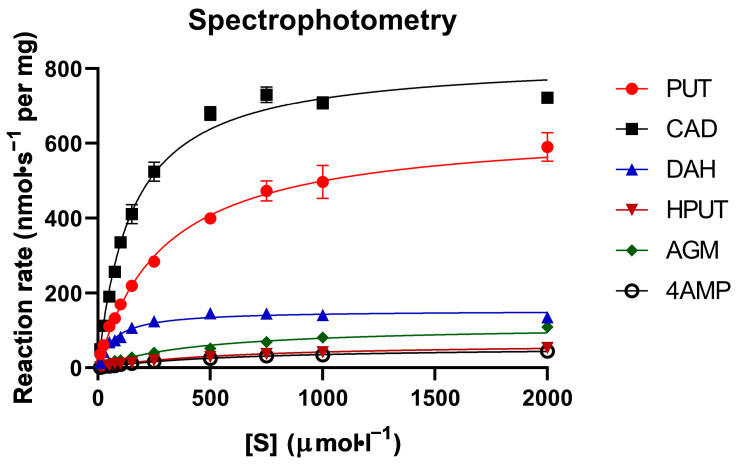
Saturation curves of PSAO reactions with the studied amino compounds (spectrophotometry). All measurements were conducted using the guaiacol method with horseradish peroxidase at pH 7.0 with varying substrate concentrations. The data presented were averaged from three independent measurements, as indicated by the error bars. The color and symbol coding explained on each graph represent individual substrates.

Six known PSAO substrates were chosen for experiments in this study. These included the natural substrates putrescine (PUT), cadaverine (CAD), and agmatine (AGM), as well as the synthetic compounds 1,6-diaminohexane (DAH), 2-hydroxyputrescine (HPUT), and 4-(aminomethyl)piperidine (4AMP). The established spectrophotometric method using horseradish peroxidase and guaiacol as reagents was chosen to obtain reference kinetic data results. These experimental data are presented in Table 1. This activity assay has repeatedly been shown to be a reliable approach [37,38]. It is also cost-effective, utilizing relatively inexpensive chemicals. Reactions with substrates were monitored at 436 nm over a period of 3 min. Triplicate absorbance values were used to calculate the kinetic parameters *K*_m_, *k*_cat_, and *k*_cat_/*K*_m_ for substrate efficiency comparisons. The amount of the enzyme added to the reaction mixture was optimized by activity assays at a 1 mmol·L^−1^ substrate concentration, which was 0.8 µg for PUT and CAD and 3.5 µg for the other substrates. The *K*_m_ values were on the order of 10^−4^ mol·L^−1^, with the lowest values found for DAH and CAD, and the highest values observed for 4AMP and HPUT. The *k*_cat_ values ranged between 4 and 61 s^−1^, with the highest values observed for CAD, PUT, and DAH, while the lowest values were found for 4AMP, HPUT, and AGM (Table 1). The efficiency constant *k*_cat_/*K*_m_ indicated that the best substrates for PSAO were CAD, PUT, and DAH, in that order. The least effective substrate was 4AMP, followed by HPUT and AGM. The corresponding experimental saturation curves are provided in Figure 1.

Similarly, MALDI measurements were initiated following optimizations. First, the potassium phosphate buffer used in the spectrophotometric assay, which is incompatible with MALDI sample preparation, was replaced by ammonium bicarbonate buffer. The volatile buffer was adjusted to pH 7.0, and the performance of the enzyme was evaluated by spectrophotometry for comparison. It was found to be unchanged with PUT and AGM as substrates. Therefore, this buffer was used in all MALDI-based analyses. It was also necessary to optimize enzyme amounts and incubation times for individual substrates to observe mass peaks with sufficient intensities. The diluted working PSAO solution contained 0.34 mg·mL^−1^ protein and was applied in the following arrangements: PUT, 3 µL—5 min; CAD 2–4 µL—3 min; DAH 3 µL—10 min; HPUT 4 µL—60 min; AGM 5 µL—20 min; and 4AMP 10 µL—10 min.

**Figure 2:**
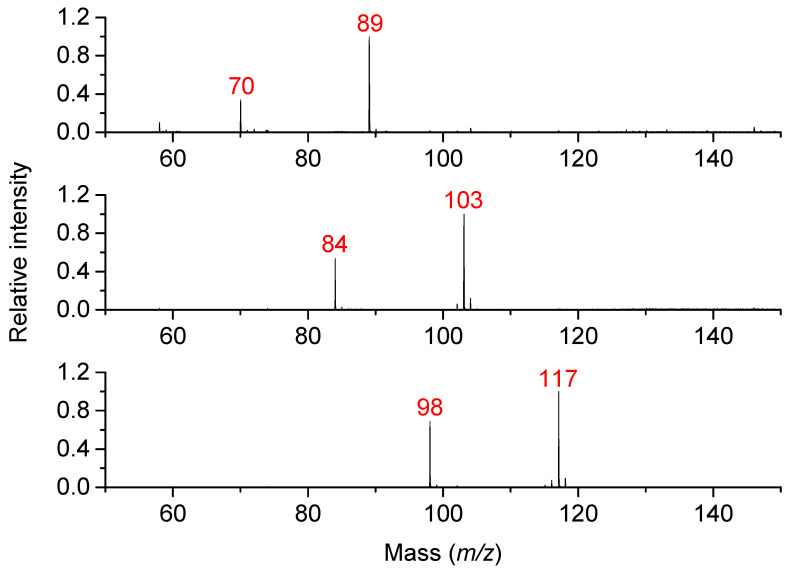
MALDI-TOF mass spectrometry of mixtures containing diamines and aminoaldehydes. The measurements were conducted to compare signal intensities. From the top: PUT and 4-aminobutanal, CAD and 5-aminopentanal, and DAH and 6-aminohexanal. The spectra were acquired using CHCA as a matrix in the presence of CTAB. The measured solutions contained 0.5 mmol·L^−1^ of each compound.

Three diamines (PUT, CAD, and DAH) and their oxidation products (4-aminobutanal, 5-aminopentanal, and 6-aminohexanal, respectively) were measured by MALDI-TOF MS in the reflector positive ion mode to compare the signal intensities at equal concentrations of 500 and 100 µmol·L^−1^ in 50 mmol·L^−1^ NH_4_HCO_3_, pH 7.0 (adjusted by acetic acid). The aminobutanal was obtained by acidic hydrolysis of commercial diethylacetal, whereas the others were synthesized by oxidation of the corresponding aminoalcohols. As shown in Figure 2, higher intensities were consistently observed for the diamines in a mutual comparison, and the aminoaldehydes were detected in their cyclic forms as follows: 1-pyrroline (3,4-dihydro-2*H*-pyrrole, C_4_H_7_N, *m*/*z* 70), 1-piperideine (2,3,4,5-tetrahydropyridine, C_5_H_9_N, *m*/*z* 84), and 3,4,5,6-tetrahydro-2*H*-azepine (C_6_H_11_N, *m*/*z* 98). The determined intensity ratios of diamine/aminoaldehyde decreased with the carbon chain length as follows: 2.9 (C_4_), 2.0 (C_5_) and 1.6 (C_6_). These ratios were used to calculate the product concentration in the PSAO reaction mixtures during subsequent kinetic experiments.

**Figure 3:**
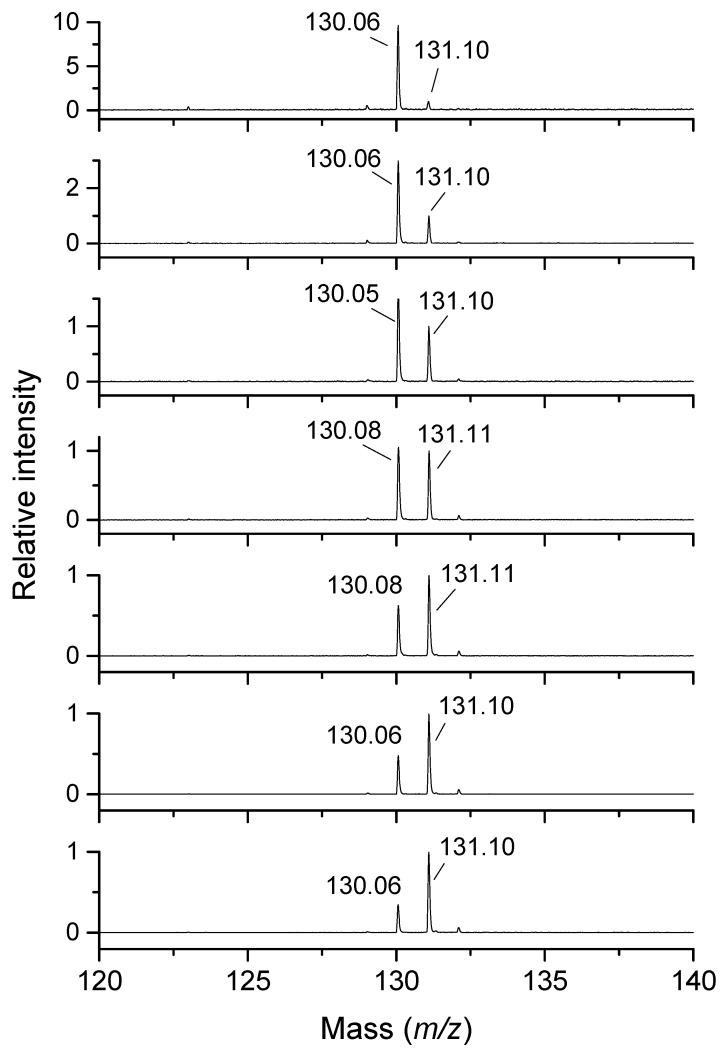
MALDI-TOF mass spectra of the PSAO reaction mixture with AGM at pH 7.0. This figure shows changes in the intensity ratio between the product 2-hydroxypyrrolidine-1-carboximidamide (*m*/*z* 130) and the substrate AGM (*m*/*z* 131). The initial substrate concentration increases from the top to the bottom (25, 50, 75, 100, 150, 200, and 250 μmol∙L^−1^). PSAO was applied in an amount of 1.4 μg, and the reaction mixture (1 mL) was incubated for 20 min. The spectra were acquired using CHCA as a matrix in the presence of CTAB.

Piperidine-4-carbaldehyde (*m*/*z* 114), the deduced product of 4AMP oxidation, displayed signal intensities that were 4.8 times lower than those of the substrate (*m*/*z* 115) at the same concentration. A similar intensity conversion factor of 4.6 was obtained for pyridine-4-carbaldehyde (*m*/*z* 108), which was also used as a possible, though non-identical, standard. *N*′-hydroxypyrrolidine-1-carboximidamide (*m*/*z* 130), a structural isomer of 2-hydroxypyrrolidine-1-carboximidamide, the unavailable oxidation product of AGM (*m*/*z* 131), provided an intensity conversion factor of 2.9. MALDI measurements with AGM are illustrated in Figure 3. For HPUT (*m*/*z* 105), the same intensity conversion factor determined with PUT and 1-pyrroline was used, assuming a similar ionization efficiency between 1-pyrroline (*m*/*z* 70) and 3,4-dihydro-2*H*-pyrrol-3-ol (4-hydroxy-1-pyrroline [39], *m*/*z* 86).

**Figure 4:**
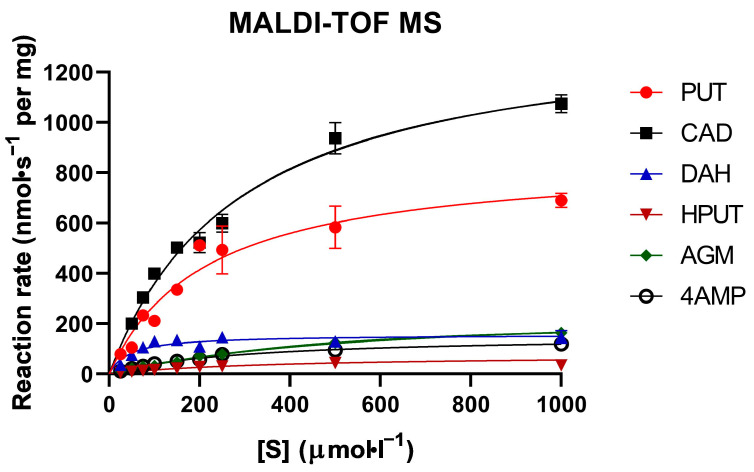
Saturation curves of PSAO reactions with the studied amino compounds (MALDI-TOF). All measurements were conducted at pH 7.0 with varying substrate concentrations. The data presented were averaged from three independent measurements, as indicated by the error bars. The color and symbol coding explained on each graph represent individual substrates.

Table 2 summarizes the results obtained using MALDI-TOF MS. The original data for CAD (including the deduction of the intensity conversion factor) and the procedure for their software processing are provided for illustration in the Supplementary File. The standard deviation for the conversion factors was around 10%, which is comparable to the standard deviations for the determination of *K*_m_ and *V* from repeated measurements. The determined *K*_m_ values were generally on the order of 10^−4^ mol·L^−1^, except for DAH, which showed the lowest value of 60 µmol·L^−1^. The highest *K*_m_ values were registered for AGM and HPUT. The *k*_cat_ values ranged between 5 and 103 s^−1^, with the highest values observed for CAD, PUT, and AGM, while the lowest values were found for HPUT, 4AMP, and DAH (Table 2). The efficiency constant *k*_cat_/*K*_m_ indicated that the best substrates for PSAO were CAD, PUT, and DAH, in that order. The least efficient substrate was HPUT followed by AGM and 4AMP. The corresponding experimental saturation curves are provided in Figure 4.

## 3. Discussion

Understanding metabolism enhances our knowledge of polyamine cellular functions and the mechanisms of related diseases, potentially leading to the development of novel therapeutic strategies. When studying biosynthetic or catabolic enzymes, metabolic research necessarily involves activity assay methods. Our aim was to contribute a novel method for determining DAO activity, specifically focusing on a principle that utilizes direct measurement of the degraded substrate and generated product via MALDI-TOF MS. PSAO has been used as a model to study the reaction mechanism, cofactors, substrates, and inhibitors of plant DAOs [16,40]. The most common activity assays for DAOs are spectrophotometric and amperometric (e.g., Clark electrode). Spectrophotometric methods typically detect the product aldehyde or hydrogen peroxide, while amperometric methods monitor oxygen consumption. Spectrophotometric assays are simple, cost-effective, and widely accessible, and they use standard lab equipment. They allow for high-throughput analysis and can be highly sensitive, with the flexibility to measure various aspects of enzyme kinetics. These advantages make them suitable for routine and large-scale studies [41]. Conversely, using an oxygen electrode to determine DAO activity offers several benefits over spectrophotometric methods. It provides direct, real-time measurement of oxygen consumption, which directly correlates with enzyme activity. The oxygen electrode is less prone to interference from colored substances or sample turbidity. Additionally, it allows for continuous monitoring, enabling precise observation of reaction kinetics [42].

The newly developed MALDI-based method was designed to combine the advantages of spectrophotometric and amperometric assays, such as direct and sensitive product detection, fast measurements on the instrument (although incubation times can be optionally as long as 60 min), and robustness, while also allowing for high throughput with low sample consumption. The selection of the matrix for MALDI measurements was based on the laboratory’s previous positive experience with measuring enzyme reaction mixtures containing low-molecular-weight compounds [31,43]. Therefore, CHCA with CTAB as an additive [36] was used in all measurements. MALDI is generally known as an ionization technique that is not suitable for quantitative measurements unless internal standards or ratiometric approaches are used [31]. This limitation is due to issues such as inhomogeneous matrix crystallization, variable ionization efficiency, limited dynamic range, and signal suppression, all of which lead to inconsistent and unreliable signal intensities. Additionally, spot-to-spot and shot-to-shot variability in desorption/ionization further complicate accurate quantification [44]. The selected set of substrates to be measured was complemented by the addition of (amino)aldehyde products from their enzymatic oxidation or similar compounds. By measuring the signal intensity ratios of the corresponding pairs at equimolar concentrations, intensity conversion factors were determined to correct for differences in ionization efficiency. In this arrangement, the aminoaldehydes (specifically, their cyclic forms after dehydration) or heterocyclic aldehydes consistently provided lower intensities than the initial diamines.

Six substrates were analyzed by spectrophotometry and MALDI-TOF MS to measure PSAO activity and determine the kinetic constants *K*_m_ and *k*_cat_. Most of these compounds have long been recognized as good substrates, and their substrate properties with DAOs from legumes have been repeatedly measured using various activity assay methods. Table 3 compares the kinetic data obtained from the literature and the BRENDA database (https://www.brenda-enzymes.org/, accessed on 31 August 2024). According to the *k*_cat_ values (considering average numbers), the highest activity is achieved with CAD and PUT. These compounds also bind well at the active site, as indicated by the relatively low *K*_m_ values (10^−5^–10^−4^ mol·L^−1^). Interestingly, DAH shows similar, if not better, binding, but its conversion is slower by almost one order of magnitude. Published data for AGM and HPUT show that they are weaker substrates. In this work, 4AMP was described as a new substrate for the first time, with reference data available only for the structurally similar compound, 4-(aminomethyl)pyridine [20]. The reference spectrophotometric results, regarding both the *K*_m_ and *k*_cat_ values, appeared in good agreement with the literature and database: compare Table 1 and Table 3. The guaiacol spectrophotometric method offers continuous measurements with a routine pipetting of samples. The measurement is short; in our case, it was up to 3 min for a single sample, and it can be multiplied and made more effective using spectrophotometers with cuvette carousels or a multicell holder. The use of stirred spectrophotometric cells was important for the reproducibility of measurements.

The present MALDI-based method was designed to be economical (with low sample consumption), robust, and capable of providing high-throughput measurements. The method was optimized using a 1 mL reaction mixture, but the volume can be downscaled to less than 50 µL. The only limitation in this regard is the reproducible addition of the enzyme in microliter amounts, which can be achieved using a repeating syringe dispenser. Pipetting enzyme aliquots into a set of pre-incubated reaction mixtures in test tubes is quick and can be performed even faster with a multichannel pipette. Before the measurements, it was necessary to ensure the formation of sufficient product intensity at the lowest substrate concentrations and then adjust the incubation period (3–60 min) and enzyme amount accordingly. The prolonged incubation periods were necessary to achieve sufficient product signal intensities for weak substrates (using the same diluted enzyme as for the best substrates), which limits the fluency and speed of the method and may increase the risk of side reactions in the reaction mixture. Adding more enzyme to the reaction mixture (to increase the substrate conversion) is a good option to overcome this limitation.

Since the sample is pipetted onto a matrix layer, pre-spotted targets are used, and again, a multichannel pipette can make the deposition more efficient. In any case, the enzymatic reaction is stopped by cooling on ice, minimizing the risk of delay and related inaccuracies. With a few exceptions, the obtained results were in agreement with the reference spectrophotometric data (see and compare Table 1 and Table 2). This underlines the importance of knowing intensity correction factors for differences in the ionization of the substrate and product. Importantly, another limiting factor of the method—the unavailability of an internal standard that is chemically identical to the reaction product—can be solved by using a structurally similar compound. In this case, it is again necessary to evaluate peak intensities in the measured mixture with an equimolar substrate to obtain an accurate intensity correction factor. Otherwise, the calculated *K*_m_ values would become inaccurately low, and similarly, the *k*_cat_ values would be underestimated. Interestingly, the *k*_cat_ values for PUT, CAD, AGM, and 4AMP were significantly higher with the MALDI method, indicating a possible underestimation of the respective spectrophotometric results. Limiting factors that can influence the efficiency and accuracy of coupled enzyme detection reactions in activity assays have been discussed in the literature for several enzymes. These factors include the complexity of the reaction mixture, the presence of inhibitors, and side reactions [46]. On the other hand, unexpected inaccuracies in MALDI assay results must also be considered, which could arise from the ionization process and the influence of signals by ion suppression and interferences.

## 4. Materials and Methods

### 4.1. Chemicals

All PSAO substrates (in the form of hydrochloride salts, with only agmatine as a sulfate salt) were purchased from Merck (Steinheim, Germany), except for dihydrochlorides of 1,6-diaminohexane and 2-hydroxyputrescine (1,4-diamino-2-butanol), which were gifts from Prof. Emeritus Lumír Macholán (Masaryk University in Brno). 4-Aminobutanal diethylacetal was from Merck. For MALDI-TOF measurements, this compound was hydrolyzed in 0.5 M HCl (10 µL of the diethylacetal was added to 0.5 mL of the acid, incubated at 100 °C for 10 min, then neutralized with ammonia and diluted to the desired concentration). 5-Aminopentanal a 6-aminohexanal were synthesized as follows: 5-aminopentanol and 6-aminohexanol (both from Merck), each 15 mmol, were first reacted with equimolar amount of di-*tert*-butyl dicarbonate in dichloromethane (50 mL) for 2 h to protect the amino group. The resulting Boc-protected products were isolated after solvent removal on a rotary vacuum evaporator. The protected aminoalcohols were converted to the corresponding protected aminoaldehydes by Swern oxidation with oxalyl chloride and dimethyl sulfoxide (DMSO) at −70 °C as described [47]. Prior to the final Boc-cleavage, the identity and purity of *N*-Boc-aminoaldehydes was briefly confirmed by NMR analysis. *N*-Boc-aminohexanal: ^1^H-NMR (400 MHz, DMSO-d_6_) δ 9.64 (t, *J* = 1.5 Hz, 1H), 6.72 (t, *J* = 4.9 Hz, 1H), 2.88 (q, *J* = 6.6 Hz, 2H), 2.39 (td, *J* = 7.3, 1.5 Hz, 2H), 1.54–1.46 (m, 2H), 1.39–1.32 (m, 4H), 1.36 (s, 9H), 1.25–1.18 (m, 2H). *N*-Boc-aminopentanal: ^1^H-NMR (400 MHz, DMSO-d_6_) δ 9.64 (t, *J* = 1.5 Hz, 1H), 6.75 (t, *J* = 4.9 Hz, 1H), 2.89 (q, *J* = 6.9 Hz, 2H), 2.41 (td, *J* = 7.2, 1.5 Hz, 2H), 1.50–1.45 (m, 2H), 1.39–1.34 (m, 4H), 1.36 (s, 9H). Hydrolysis to remove the protecting group was carried out in 0.5 M HCl at 70 °C for 10 min. Horseradish peroxidase, 4-(aminomethyl)piperidine, 4-pyridine carbaldehyde, 1-Boc-piperidine-4-carbaldehyde (deprotected before use as described above), and *N*′-hydroxypyrrolidine-1-carboximidamide hydrochloride were all purchased from Merck. All other chemicals were of analytical purity grade.

### 4.2. Purification of Pea Seedling Amine Oxidase

PSAO was purified to homogeneity by a modified procedure that combined precipitations and liquid chromatographic steps conducted at 5 °C [37,48]. Etiolated pea seedlings (7 days old, 1 kg), with roots and cotyledons removed, were homogenized in 2 L of 0.1 mol·L^−1^ potassium phosphate buffer, pH 7.0, containing 10% (*w*/*v*) sucrose, 15 mmol·L^−1^ mercaptoethanol, and 1 mmol·L^−1^ EDTA. The homogenate was centrifuged repeatedly (4100× *g*), and the supernatants were subjected to sequential precipitation with (1) protamine sulfate (added as a 5% suspension at a weight ratio of 1:10 relative to the supernatant protein), (2) manganese chloride (added as 0.5 mol·L^−1^ solution to achieve a final concentration of 7.5 mmol·L^−1^ MnCl_2_), and (3) 65% saturated ammonium sulfate. The final precipitate was dissolved in 20 mmol∙L^−1^ potassium phosphate buffer, pH 6.0, containing 5 mmol∙L^−1^ 2-mercaptoethanol, 1 mmol∙L^−1^ EDTA, and 5% (*w*/*v*) glycerol (buffer A), to a total volume of 40 mL.

The enzyme solution, after centrifugation at 4100× *g* to remove sediments, was desalted on a Sephadex G-25 column (2.5 × 50 cm) in buffer A at a flow rate of 2 mL·min^−1^, monitored by absorbance at 280 nm. It was then applied to a DEAE-Sepharose column (2.5 × 20 cm) in buffer A at 2 mL·min^−1^. The flow-through protein fraction was loaded onto a hydroxyapatite column (2.5 × 20 cm), equilibrated in 20 mmol∙L^−1^ potassium phosphate buffer, pH 7.0 (buffer B), at 2 mL·min^−1^, and eluted with 0.5 mol∙L^−1^ potassium phosphate buffer, pH 7.0 (monitored at 280 nm). After overnight dialysis against 20 mmol∙L^−1^ potassium phosphate buffer, pH 5.8 (buffer C), the partially purified PSAO was concentrated by ultrafiltration and further separated by two steps of medium-pressure chromatography on a Bio-Logic Duo Flow chromatograph (Bio-Rad, Hercules, CA, USA).

First, the enzyme solution was loaded onto a UNO S12 column (Bio-Rad) in buffer C at 2 mL·min^−1^ and eluted by a programmed gradient of 0–1 mol∙L^−1^ NaCl in buffer C (monitored at 280 and 500 nm). The eluted enzyme fraction, checked for activity by the spectrophotometric assay (see below), was dialyzed overnight against buffer B, and concentrated by ultrafiltration. The final step involved gel permeation chromatography on an ENrich SEC650 column (Bio-Rad) in buffer B at a flow rate of 0.75 mL·min^−1^ (monitored at 280 and 500 nm). The final enzyme preparation exhibited a specific activity of 550 nkat· mg^−1^. The purification grade and activity yield were 210 and 37%, respectively, as measured spectrophotometrically with PUT as a substrate at a concentration of 2.5 mmol·L^−1^.

### 4.3. Spectrophotometric Activity and Protein Assays

PSAO activity was assayed by monitoring hydrogen peroxide production at 30 °C using a modified spectrophotometric method with guaiacol and horseradish peroxidase [24]. The reaction mixture contained 0.1 mol·L^−1^ potassium phosphate buffer, pH 7.0, 0.5 mmol·L^−1^ guaiacol, 2 μg of horseradish peroxidase (140 U·mg^−1^), an appropriate amount of pure enzyme (0.8–3.5 µg), and substrate, in a total volume of 1.50 mL. The stock solution of the enzyme in 20 mmol∙L^−1^ potassium phosphate buffer, pH 7.0, contained 0.3–0.7 mg∙L^−1^ protein. The substrate was added as the final component (50 μL) to initiate the reaction. Its final concentration ranged between 10 μmol·L^−1^ and 2.5 mmol·L^−1^. The reaction was monitored over time at 436 nm, using an extinction coefficient of 4500 mol^−1^·L·cm^−1^ for calculations [49]. Protein concentration was determined using the Bradford spectrophotometric method [50] with bovine serum albumin as a standard.

### 4.4. MALDI-TOF Mass Spectrometry

PSAO reaction mixtures (1 mL) in test tubes were prepared in 50 mmol·L^−1^ NH_4_HCO_3_ adjusted to pH 7.0 by acetic acid. Substrate concentrations ranged between 25 and 1000 μmol∙L^−1^, and the solution was preincubated in a thermostat at 30 °C. The reaction was initiated by adding the enzyme (1.0–3.5 µg, based on substrate performance evaluated in the spectrophotometric measurements) and proceeded at 30 °C and 500 RPM for 3–60 min. The reaction was stopped by cooling on crushed ice prior to further analysis. All MS measurements were performed using a Microflex LRF20 MALDI-TOF mass spectrometer equipped with a 60 Hz nitrogen laser (λ_max_ = 337 nm). The instrument was set to reflector mode and operated using the flexControl 3.4 spectra acquisition software (Bruker Daltonik, Bremen, Germany). The parameters of the instrument were as follows: IS1 voltage of 19 kV, IS2 voltage of 16.2 kV, lens voltage of 8.9 kV, reflector voltage of 20 kV, and detector voltage of 1650 V. Delayed extraction was employed with the pulsed ion extraction time set to 150 ns. The matrix used was 0.1 mol·L^−1^ CHCA (Bruker Daltonik) in acetone–water, 4:1, *v*/*v*, containing 0.1 mmol·L^−1^ CTAB [36]. Initially, 1 μL aliquots of the matrix were deposited in sample positions on an MSP BigAnchor 96 BC target plate (Bruker Daltonik) and left to dry at ambient temperature. Reaction mixture samples (1 μL) were then applied onto the crystallized matrix and left to dry. Mass spectra were accumulated from 500 to 1000 single-pulse shots. Matrix peaks at *m*/*z* 190 (CHCA), 284 (cetrimonium), and 379 (CHCA) were used for internal calibration. Mass spectra were evaluated using flexAnalysis 3.4 (Bruker Daltonik).

### 4.5. Kinetic Data Processing

Numerical data from spectrophotometry (absorbance values) and MALDI-TOF MS (peak intensities) were processed using calculations in Microsoft Excel 2016 to obtain reaction rate values. For the MALDI-based method, substrate conversion over time was calculated from peak intensities using the formula I_p_/(I_p_ + I_s_), where I_p_ represents product signal intensity and I_s_ represents substrate signal intensity. These intensity ratios were then used to calculate the product amount in the reaction mixture, in order to express the reaction rate. Product signal intensities with pure compounds were evaluated in the presence of equal substrate amounts to determine intensity correction factors accounting for better ionization of the substrate at the same concentration. Michaelis–Menten graphs were plotted and analyzed using GraphPad Prism 8.0.1 software to determine the kinetic parameters *K*_m_ and *V* (the latter was used for *k*_cat_ calculation).

## 5. Conclusions

We have demonstrated that it is possible to measure the enzyme activity of DAOs by means of MALDI-TOF MS following a ratiometric approach. After the necessary initial optimization of the enzyme amount in the reaction mixture and incubation time, reliable and reproducible data can be obtained. The measurements are simple and applicable for determining kinetic parameters. For accuracy, it is important to evaluate differences in the ionization of the substrate and product and to use intensity correction factors in data processing calculations.

## Figures and Tables

**Table 1:** Kinetic parameters of the PSAO reaction with amine substrates (spectrophotometry). The results were obtained by spectrophotometry using a coupled reaction with guaiacol and horseradish peroxidase. The *K*_m_ and *V* values were calculated from the measured absorbance data using GraphPad Prism 8.0.1 software. All data series were analyzed in triplicate.

Substrate	*K*_m_ [μmol·L^−1^]	*V* [nmol·s^−1^ per mg]	*k*_cat_ [s^−1^]	*k*_cat_/*K*_m_ [mol^−1^·L·s^−1^]	*k*_cat_/*K*_m_ [Relative]
PUT	289 ± 15	644 ± 12	47.4	1.64 × 10^5^	1.000
CAD ^1^	148 ± 8	827 ± 13	60.9	4.11 × 10^5^	2.506
DAH ^1^	95 ± 7	176 ± 6	13.0	1.37 × 10^5^	0.835
HPUT	589 ± 33	67 ± 2	4.9	8.32 × 10^3^	0.051
AGM	446 ± 23	114 ± 3	8.4	1.88 × 10^4^	0.115
4AMP	618 ± 24	57 ± 1	4.2	6.80 × 10^3^	0.041

^1^ Excess substrate inhibition observed at more than 500 µmol·L^−1^ concentrations.

**Table 2:** Kinetic parameters of the PSAO reaction with amine substrates (MALDI). The results were obtained by MALDI-TOF mass spectrometry. The *K*_m_ and *V* values were calculated from the measured product and substrate peak intensity ratio data using GraphPad Prism 8.0.1 software. All data series were analyzed in triplicate.

Substrate	*K*_m_ [μmol·L^−1^]	*V* [nmol·s^−1^ per mg]	*k*_cat_ [s^−1^]	*k*_cat_/*K*_m_ [mol^−1^·L·s^−1^]	*k*_cat_/*K*_m_ [Relative]
PUT	214 ± 32	859 ± 54	63.2	2.95 × 10^5^	1.000
CAD	289 ± 23	1401 ± 51	103.0	3.56 × 10^5^	1.207
DAH ^1^	56 ± 18	175 ± 21	12.9	2.30 × 10^5^	0.780
HPUT	379 ± 38	77 ± 5	5.7	1.50 × 10^4^	0.051
AGM	576 ± 44	261 ± 11	19.2	3.33 × 10^4^	0.113
4AMP	286 ± 21	153 ± 5	11.3	3.95 × 10^4^	0.134

^1^ Excess substrate inhibition observed at more than 500 µmol·L^−1^ concentrations.

**Table 3:** The kinetic parameters *k*_cat_ and *K*_m_ as described in the literature [37,38,45] and the BRENDA database for DAOs from legumes.

Substrate	Published *k*_cat_ Values [s^−1^]	Published *K*_m_ Values [µmol·L^−1^]
PUT	5–280	65–430
CAD	5–500	60–400
DAH	20–40	90–160
HPUT	1–20	250–740
AGM	1–45	150–560
4AMP ^1^	n.a.	n.a.

^1^ For a structurally similar compound, 4-(aminomethyl)pyridine, the *k*_cat_ and *K*_m_ values were 3 s^−1^ and 100 μmol∙L^−1^, respectively, for the enzyme from *Lathyrus cicera* at pH 7.2 [20]; n.a. stands for “not available”.

## Data Availability

The data that support the findings of this study are available from the corresponding author, M.Š., upon reasonable request.

## Supplementary Materials

The following supporting information can be downloaded at https://www.mdpi.com/article/10.3390/molecules29204878/s1: Table S1: Cadaverine and piperideine: a comparison of signals in MALDI-TOF mass spectra at their equimolar ratios; Table S2: MALDI-TOF MS of pea seedling amine oxidase reaction mixtures: evaluation of cadaverine and piperideine signals; Table S3: Excel evaluation of experimental data; Table S4: GraphPad Prism evaluation of experimental data.

## Abbreviations

AGM, agmatine; 4AMP, 4-(aminomethyl)piperidine; CAO, copper amine oxidase; CAD, cadaverine; CHCA, α-cyano-4-hydroxycinnamic acid; CTAB, cetrimonium bromide; DAH, 1,6-diaminohexane; DAO, diamine oxidase; DMSO, dimethyl sulfoxide; HPUT, 2-hydroxyputrescine; MALDI-TOF, matrix-assisted laser desorption/ionization time-of-flight; MS, mass spectrometry; PSAO, pea (*Pisum sativum*) seedlings amine oxidase; PUT, putrescine; TPQ, topaquinone.
